# Protocol for a multicenter prospective cohort study evaluating sport activity and development of femoroacetabular impingement in the adolescent hip

**DOI:** 10.1186/s12891-020-03220-6

**Published:** 2020-04-11

**Authors:** Axel Öhlin, Nicole Simunovic, Andrew Duong, Olufemi R. Ayeni, PREVIEW investigators, Olufemi R. Ayeni, Nicole Simunovic, Andrew Duong, Yan Sim, Lehana Thabane, Matthew Skelly, Ajay Shanmugaraj, Diane Heels-Ansdell, Lisa Buckingham, Volker Musahl, Vasco V. Mascarenhas, Etienne L. Belzile, Sylvie Turmel, Rintje Agricola, Seung-Hoon Baek, Ae-Sun Chang, Axel Öhlin

**Affiliations:** Department of Surgery, Division of Orthopaedic Surgery, McMaster University, 1200 Main St West, 4E15, Hamilton, ON L8N 3Z5 Canada

**Keywords:** Femoroacetabular impingement, Magnetic resonance imaging, Sport specialization, Adolescents

## Abstract

**Background:**

Femoroacetabular impingement (FAI) is an important cause of hip pain in young and active individuals and occurs as a result of size and shape mismatch between the femoral head and acetabulum. Open physes in children can make hips more susceptible to injury, and high impact forces have been suggested to affect the developing femur. The diagnosis of FAI has recently risen, especially within adolescent populations, and there is an increasing trend towards year-round participation in sports with early specialization. The PREVIEW study is an international longitudinal study designed to determine the association between sport specialization in adolescence and the development of hip impingement.

**Methods:**

This is a multicentre prospective cohort study evaluating 200 participants between the ages of 12–14 that include sport specialists at the moderate to vigorous physical activity (MVPA) level and non-sport specialists at any activity level. We will monitor physical activity levels of all participants using an activity log and a wrist-mounted activity tracker, with synced data collected every 3 months during the study period. In addition, participants will be evaluated clinically at 6, 12, and 18 months and radiographically at the time of enrolment and 24 months. The primary outcome is the incidence of FAI between groups at 2 years, determined via MRI. Secondary outcomes include hip function and health-related quality of life between subjects diagnosed with FAI versus no FAI at 2 years, as determined by the Hip Outcome Score (HOS) and Pediatric Quality of Life (PedsQL) questionnaires.

**Discussion:**

It is important to mitigate the risk of developing hip deformities at a young age. Our proposed prospective evaluation of the impact of sport activity and hip development is relevant in this era of early sport specialization in youth. Improving the understanding between sport specialization and the development of pre-arthritic hip disease such as FAI can lead to the development of training protocols that protect the millions of adolescents involved in sports annually.

**Trial registration:**

PREVIEW is registered with clinicaltrials.gov (NCT03891563).

## Background

**Femoroacetabular impingement (**FAI) can cause hip pain and may lead to the development of osteoarthritis of the hip later in life. Some cross-sectional studies have estimated that the prevalence of hip impingement ranges from 14 to 17% among asymptomatic young adults to almost 95% among competitive athletes [1–3]. FAI occurs as a result of a size and shape mismatch between the femoral head and the acetabulum. FAI is typically classified into 2 subtypes; cam-type (a misshaped femoral head) or pincer type (an over covered or deep socket). Most adult patients (18+ years) have a combination of both types of impingement [4]. With FAI, the abnormal femoral head and acetabular rim of the hip joint collide or “impinge” during movements such as hip flexion and rotation [5]. Typically, patients with this condition experience hip pain and loss of hip function. The development of hip pain in this manner serves as an indicator for early cartilage and labral damage, potentially resulting in hip osteoarthritis [5].

The incidence of FAI has recently risen across all age groups, but it has been especially notable within paediatric, or more specifically, adolescent populations [6]. In the adult, FAI is most commonly attributed to an “idiopathic anatomic variant” [6]. In the paediatric population, implicated causes of FAI have included genetics, subclinical paediatric hip disease, and stresses applied to the hip joint from certain high-intensity sports [7]. According to Packer et al., there is no definitive evidence that FAI is transmitted genetically, and in otherwise healthy children, there is growing evidence that FAI has a higher prevalence in athletes who performed at a high level during adolescence [8–11].

High impact activities in combination with intensity of various kinds have been shown to affect the developing femur [12]. Among children, open physes and growing cartilage make them more susceptible to injury and shear forces that can result in premature physeal arrest, apophyseal avulsion fractures, and chondral injuries [13]. A higher prevalence of cam deformities (> 50%), both symptomatic and asymptomatic, has been shown in adolescent athletes that play ice hockey, basketball, and soccer when compared to controls that did not play sports [7, 10, 11, 14–17]. These sports involve repetitive deep flexion, flexion-adduction or extension-abduction movements, which bring the cam lesion on the femoral head or the pincer lesion on the acetabulum into conflict [6]. Therefore, participating in high impact sports during growth likely plays an important role in the development of hip deformities. This is concerning given: a) the increasing trend toward year-round participation in youth sports with early specialization and b) FAI has been shown to predispose patients to the progression of hip osteoarthritis [18–21]. Still, most studies in the current literature that evaluate the relationship between sports and the development of FAI are relatively small, retrospective case-controls and the most recent evidence demonstrates conflicting results regarding how and when primary FAI develops in relation to skeletal maturity [7, 22]. Accordingly, the current literature notes the need for longitudinal or prospective magnetic resonance imaging (MRI) studies to understand the etiology of primary FAI development to identify preventive strategies, delineate radiographic values, define specific indications for operative management, and examine long-term outcomes to determine optimal management [7, 12].

### Study objectives

The primary research objective is to determine if sport specialization at the moderate-to-vigorous physical activity (MVPA) level [23–27] is associated with the development of both symptomatic and asymptomatic FAI (i.e. cam, pincer, or mixed-type) in adolescents (ages of 12–14 years) at 2 years post-enrollment determined via MRI.

The secondary research objectives are to determine if the presence of FAI is associated with changes in hip function and health-related quality of life (HRQL) at 2 years post-enrollment determined via the Hip Outcome Score (HOS) and Pediatric Quality of Life (PedsQL) questionnaires.

## Methods

### Study design

This is a longitudinal cohort study of 200 participants between the ages of 12 and 14 (i.e. critical age of development of the femoral head and neck). Participants will be evaluated clinically at enrollment and 6, 12, 18, and 24 months and radiographically (with MRI) at baseline and 24 months. Activity will be tracked every 3 months from enrollment to final follow-up at 2 years. Participants will be recruited from experienced hip surgeons and sports medicine researchers at multiple international clinical sites. We will observe the incidence of radiographic FAI, as determined by MRI of the hip, and evaluate hip function and HRQL using the HOS and PedsQL, respectively. Ethics approval for this study was granted by the Hamilton Integrated Research Ethics Board (Version D1.0, 1-August-2019, HIREB #7829). Any protocol amendments will be submitted to HIREB and communicated to all participating sites for submission their site-specific ethics boards.

### Participant selection

#### Eligibility criteria

The inclusion criteria are: 1) asymptomatic males and females between the ages of 12–14 years; 2) ability to speak, understand, and read the language of the clinical site; and 3) provision of informed child assent (if applicable) and parental consent.

The exclusion criteria mainly preclude known confounding factors that would impede the evaluation of the etiology of a hip deformity. These include: 1) hip is mature (i.e. closed physes) based on the baseline MRI scan; 2) hip complaints or pain in the hip; 3) previous trauma to the hip; 4) previous surgery on the hip; 5) significant medical co-morbidities (requiring daily assistance for activities of daily living); 6) history of or ongoing paediatric hip disease (Legg-Calve-Perthes; slipped capital femoral epiphysis, hip dysplasia); 7) participants that have contraindications and/or are unwilling to undergo an MRI (e.g. claustrophobia); 8) participants who will likely have problems, in the judgment of the investigator, with maintaining follow-up; and 9) any other reasons the investigator feels is relevant for excluding the subject (e.g. participant is planning to move in the next 2 years, unreliable access to transportation to attend follow-up visits, etc.).

#### Participant recruitment and screening

The study activities at each participating clinical site will be led by experienced hip surgeons and sports medicine physician researchers. However, because we are looking for healthy volunteers, participants might not be exclusively recruited from the co-investigators’ clinics. Instead, we will use several recruitment strategies that have proven successful in our pilot study. These include: 1) posting study flyers throughout each participating institution; 2) distributing study flyers to grades in the eligible age range at school boards in the regions of the recruiting institutions; and 3) having the co-investigators reach out to community coaches and competitive sport organizations and clubs, and presenting the study to the organizers, parents, and adolescent athletes. Potentially eligible subjects will be approached along with their parents/guardians for consent for participation.

### Study exposure

The study groups will be defined by ‘exposure’ to varying levels of physical activity (i.e. exposure variables) and include sport specialists at the MVPA level (group 1) and non-sport specialists at any activity level (group 2).

#### Defining sport specialization

Sport specialization will be defined according to the American Orthopaedic Society for Sports Medicine (AOSSM) and American Medical Society for Sports Medicine (AMSSM) early sport specialization criteria (Table 1) [28, 33]. At the baseline/enrollment visit, we will collect information about any current sport participation, including type(s) and frequency, for each participant. One week prior to each follow-up visit, we will ask participants and their parent(s) to complete a daily activity log to record all types of sport activity the participant engaged in (e.g. games, practices, both organized and unorganized), or lack thereof.

**Table 1:** Definition of study groups according to objective measurement and guideline criteria

Study group	AOSSM criteria [28]	Activity tracker criteria	Duration
Group 1: Sport specialist, MVPA	1. Participation in intensive training and/or competition in organized sports greater than 8 months per year (essentially year round) [29] 2. Participation in **1 sport** to the exclusion of participation in other sports (limited free play overall) [30] 3. Involving prepubertal (seventh grade or roughly age 12 years) children.	Greater than 180 accumulated minutes of MVPA [31, 32] during participation in one sport type across one week of activity tracking	Meets criteria within either one or both years of follow-up
Group 2: Non-sport specialist, any activity level	1. Participation in **more than 1 sport** at any physical activity level **OR** 2. Participation in **none or low** training and/or competition in organized sports for any period of time. 3. Involving prepubertal children.	Greater than or less than 180 accumulated minutes of MVPA across one week of activity tracking	Meets criteria during both years of follow-up

*AOSSM* American Orthopaedic Society for Sports Medicine, *MVPA* moderate-to-vigorous physical activity

#### Defining activity level

We will cross-reference the activity log with data from an objective wrist-worn activity tracker (Garmin® Vivofit 4) to define sport and other activity intensity [34]. The Vivofit is a small, lightweight, waterproof activity tracker with high validity (relative to reference measures of physical activity and direct observation), high reliability, low reactivity in children, and has been used successfully in multiple paediatric physical activity studies [26, 35–37]. The Vivofit utilizes accelerometer measurements to calculate the Metabolic EquivalenTs (METs) of energy expenditure, where activity greater than 3 METs corresponds to MVPA. The categorical activity output includes data for sedentary, fairly active, and MVPA levels. Relevant data also includes duration of MVPA and number of total active minutes per day [38]. We will use the cut-off of ≥180 min [31, 32] of MVPA per week detected by the tracker during recorded sport participation (as per the activity log) to define the MVPA level in group 1 (Table 1). The use of the activity tracker, in conjunction with self-reported activity logs has been shown to be accurate in estimating energy expenditure in adolescents [39, 40].

One week following the baseline visit and 1 week prior to each follow-up visit, participants will wear the tracker on their wrist for a full week and to sync it with a paired tablet or cellular phone. Measuring total ‘volume’ physical activity at several time points by one-week intervals is a standard methodology used in several paediatric physical activity studies [25–27]. The combination of the activity measures and criteria described above will be used to define the study groups as per Table 1. The study groups will be defined at the completion of follow-up given participant activity levels may change over time.

### Study outcomes

#### Primary outcome

The primary outcome is the incidence of radiographic FAI between groups at 2 years, as determined by the dedicated MRI of the hip (Table 2). We will identify both symptomatic and asymptomatic cases, where asymptomatic FAI can become symptomatic in young adulthood, and/or could be associated with idiopathic osteoarthritis later in life [41].

**Table 2:** Criteria for defining FAI types

FAI type	Criteria
Cam	1. Alpha angle is > 55°; 2. Anterior head-neck offset < 10 mm; 3. Anterior head-neck offset ratio < 0.15 *Must meet 2/3 criteria to be considered cam impingement
Pincer	1. Acetabular depth < 3 mm; 2. Lateral centre-edge angle > 39°; 3. Acetabular anteversion (in the upper third of the femoral head) < 0° *Must meet 2/3 criteria to be considered pincer impingement
Mixed	*Must meet 2/3 criteria each for cam and pincer impingement (as per above) to be considered mixed impingement

*FAI* Femoroacetabular impingement

#### Secondary outcomes

Secondary outcomes include: 1) hip function and 2) HRQL between subjects with and without any type of FAI at 2 years, as determined by the HOS and PedsQL questionnaires [42, 43].

#### Outcome measures

Participants will complete the study questionnaires and undergo a physical examination (at baseline and 6, 12, 18, and 24 months) and MRI (at baseline and 24 months) of their dominant hip. Participants will sync their activity tracker data and send their activity log (at baseline and every 3 months until final follow-up at 2 years) (Table 3).

**Table 3:** Schedule of events for the PREVIEW study

Data collection	Enrollment	3 M	6 M	9 M	12 M	15 M	18 M	21 M	24 M
Screening and informed consent	●								
Enrolment data (Demographics)	●								
Physical hip examination	●		●		●		●		●
MRI	●								●
Follow-up form	●		●		●		●		●
Activity tracker wear & synchronization	*	x	x	x	x	x	x	x	x
Activity log	*	x	x	x	x	x	x	x	x
Hip Outcome Score (HOS)	●		●		●		●		●
Pediatric QoL Inventory (PedsQL)	●		●		●		●		●
Adverse events			●		●		●		●

*M* months, *MRI* Magnetic Resonance Imaging, * = 1-week post-enrollment/baseline visit, x = 1-week prior to visit

The physical examination will include range of motion (ROM) measurements in all planes documented in the supine and prone positions by a blinded research assistant (using a goniometer), as well as response of provocative hip tests on both hips [44]. Symptomatic FAI in adolescents is typically characterized by anterior hip pain aggravated by flexion activities, decreased hip internal rotation, and a positive impingement sign [7]. We will define symptomatic FAI using the following criteria: 1) positive impingement with groin pain based on flexion, adduction, and internal rotation (FADIR) test, and 2) decreased ROM: internal rotation of the hip when compared to the contralateral side by 5 degrees [45, 46].

We will use a non-contrast three dimensional (3D)-volumetric interpolated breath-hold examination (VIBE) sequence MRI [47]. This protocol has been used to document FAI-related morphology and eliminates the use of intra-articular contrast injections. All MRI and physical examination information will be provided to an independent radiologist adjudicator to determine the incidence of cam, pincer, or mixed-type FAI according to the pre-specified criteria (Table 2). We will also document the change in cartilage volume, labral pathology, herniation pits on the femoral neck, and the extent of physeal closure as these variables may predict future hip osteoarthritis [44].

The HOS is a self-administered questionnaire designed to capture hip function with clinimetric evidence for use in patients with FAI or labral tears [34, 36, 37]. The HOS was developed for young adults and has been shown to be one of the only hip function questionnaires appropriate for use in adolescents [36, 37, 48]. The PedsQL is a validated and responsive measure of HRQL in children [43]. The PedsQL was specifically designed to measure the core health dimensions outlined by the World Health Organization (physical, emotional, and social functioning), which will ensure generalizability across our global sites [49]. Adult and child versions of the HOS and PedsQL will be completed by a parent and participant at each visit. Adverse events, defined as any symptom, sign, illness, or experience that develops or worsens in severity during the course of this study, will also be documented (Table 3).

### Study follow-up

We will follow participants for 2 years as this timeframe is expected to coincide with the critical period of physeal closure in our study population. In-person follow-up visits will occur at baseline (i.e. time of enrollment), and at 6, 12, 18, and 24 months post-enrollment. Leading up to each follow-up visit, we will ask each participant to wear their activity tracker for 1 week and maintain a daily activity log. At the follow-up visit, we will collect the activity data collected in the week prior, perform a physical hip examination, and ask participants to complete the function and HRQL questionnaires. We will have 4 additional remote activity monitoring visits at 3, 9, 15, and 21 months. Each participant will undergo an MRI of their dominant hip at the baseline and 24-month visits. We designed the follow-up schedule to track activity every 3 months, maintain regular contact with the participants to help minimize loss to follow-up, and at the same time, allow enough time between in-person visits to minimize participant burden.

### Sample size calculation

We have based our sample size calculation on our pilot study data (*N* = 53), where the primary outcome event rate (incidence of FAI) is 40% for the sport specialists at the MVPA level (group 1) and 15% for the non-sport specialists at all physical activity levels (group 2). These proportions very closely match prior cross-sectional research that evaluated the incidence of FAI among athletes [10]. Because we have looked at early data from the pilot study to inform our calculation, we have adjusted our type I error rate. To achieve 80% power for a two-sided test at alpha = 0.01, we will require a sample size of 81 in each exposure group (Table 4). Given that some participants’ activity levels may change during the course of the study (where they may change from sport specialist to non-specialist, or vice versa), we will increase the sample size by 10% to ensure we have at least 81 participants in each exposure group. We will also account for a 5% rate of ineligibility (due to a mature hip [closed physes] found on the baseline MRI) and a 10% loss to follow-up rate. Therefore, we require a total sample size of 200 participants. The pilot participants will be rolled into the definitive study.

**Table 4:** Per group sample sizes. 80% power, alpha = 0.01, 2-sided testing

	FAI risk in non-sports specialists		
	5%	15%	25%
**FAI risk in sports specialists**			
20%	126	1388	1668
30%	61	193	1902
40%	38	81	240
50%	26	46	94

### Statistical plan

#### Primary analysis

As previously described, study groups will be defined at the completion of follow-up given participant activity levels may change over time. The proportion of subjects developing FAI in the sport specialist group versus non-sport specialist group will be compared using a logistic regression analysis with FAI as the dependent variable and will include the following independent variables: activity level (sport specialist versus non-sport specialist), body mass index (BMI), and sex. Results will be reported as odds ratios (OR) with 95% confidence intervals and associated *p*-values.

#### Secondary analyses

We will perform independent samples t-tests to test for differences in 24-month HOS and PedsQL scores between participants who do and do not develop FAI within the 24-month follow-up period. These analyses will be repeated comparing participants who do and do not develop symptomatic FAI. Each secondary outcome will be quantified using descriptive statistics and 95% confidence intervals. We will use multiple imputation to handle missing data.

#### Subgroup/sensitivity analyses

To assess whether the magnitude of the effect of sport activity on the development of FAI is significantly different in males than in females, we will add an interaction term between sex and sport specialist versus non-sport specialist to the primary logistic regression model. We hypothesize that sport specialization will be more strongly associated with incidence of FAI in males than in females.

No interim analyses are planned. All tests will be 2-sided with α = 0.01. We will use SAS 9.4 (Cary, NC) to perform all analyses.

### Study committees and monitoring

**Fig. 1:**
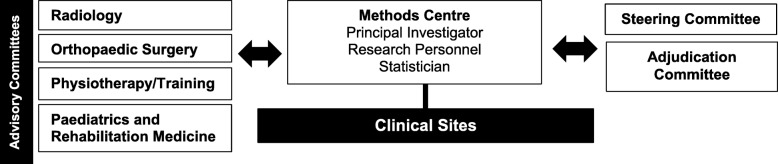
PREVIEW Study Organization

The Steering Committee will provide guidance and direction to the overall study. Specific responsibilities of the Steering Committee include reviewing and approving the study protocol and working together to resolve any challenges that arise during the study. The Steering Committee will also be supported by several Advisory Committees including: radiology, orthopaedic surgery, physiotherapy/training, and paediatrics and rehabilitation medicine. These Advisory Committees consist of individuals who are experts within their fields and have committed time to provide advice to the Steering Committee throughout the initiation and conduct of this study. An independent adjudication committee blinded to each participant’s activity level, will determine the incidence of cam, pincer, or mixed-type FAI (primary outcome) and evaluate all adverse events reported during the study. Ongoing remote and in-person clinical site monitoring will be performed by experienced Methods Centre personnel (Fig. 1).

### Data management

The case report forms (CRFs) will be the primary data collection tool for the study. All data requested on the CRF must be recorded. An Electronic Data Capture (EDC) system (iDataFax) will be used to submit data to the Methods Centre located at McMaster University. Upon receipt of the data, the personnel at the Methods Centre will make a visual check of the data and they will query all missing data, implausible data, and inconsistencies.

### Ethics and dissemination

Any subjects who meet all eligibility criteria should be approached to discuss participation in the study by someone on the study team who is knowledgeable about the study. Study personnel must approach the subject and their parent(s)/legal guardian about the study in accordance with ethical requirements for consenting children into research studies. The study protocol, clinical site-specific informed consent forms, and any participant recruitment material will need to be reviewed and approved by each clinical site’s local Ethics Board.

Information about study participants will be kept confidential and stored securely at each clinical site, only accessible to study personnel. Study records will be identified only by a coded participant number, and all records that contain participants’ names or other identifying information will be stored separately. All local databases used for storage of study data will be password protected.

## Discussion

The rationale for the PREVIEW study includes: 1) The number and diagnoses of FAI has recently risen and is especially notable within the adolescent populations; 2) High impact and high intensity activity common in many sports have the potential to cause hip damage, especially during physeal closure in the young; 3) There is an increasing trend toward year-round participation in youth sports with early specialization; and 4) An uncertainty regarding “how much is too much” of the same sport activity.

The PREVIEW study is one of the largest prospective cohort studies using MRI to determine the relationship between FAI development during skeletal maturation and physical activity. The eligibility criteria and follow-up period will help to ensure we evaluate the effect of sport activity during the critical phase of hip development and maturation. The sample size calculation is based on actual pilot data to ensure statistical power to detect differences in the incidence of FAI, hip function and HRQL among groups. Outcome assessment bias will be minimized by the use of an objective assessment tool for physical activity as well as independent adjudication of the primary outcome. The feasibility of the study has been demonstrated by the successful completion of the pilot study enrollment.

As FAI is diagnosed most frequently in athletes, and it is estimated that 30 to 45 million children and adolescents age 6–18 years old are involved in sports in the US alone, it is becoming imperative to identify factors that may predict its development, study treatments, and improve outcomes. Considering that research has demonstrated a relationship between FAI and osteoarthritis in adulthood, potentially leading to the need for total hip replacement, it has now become critical to mitigate the risk of developing these hip deformities at a young age [6]. Within organized sports, there is a current trend among coaches and parents to have children focus on one sport from a young age (i.e. sport specialization), with the thought that this dedication will allow them to reach an elite level [28, 33]. If sport specialization is associated with the development of FAI in adolescents, then there is an important opportunity to evaluate and implement improved screening and prevention strategies as well as early intervention options. A prospective evaluation of the impact of sport specialization across all types of sport during the period of hip maturation is needed in order to identify, and subsequently protect, the thousands of young people that may be at risk of developing this condition.

## Data Availability

Not applicable.

## Abbreviations

3D: Three dimensional
AMSSM: American Medical Society for Sports Medicine
AOSSM: American Orthopaedic Society for Sports Medicine
BMI: Body Mass Index
CRF: Case Report Form
EDC: Electronic Data Capture
FADIR: Flexion, ADduction, and Internal Rotation
FAI: Femoroacetabular Impingment
HOS: Hip Outcome Score
HRQL: Health-Related Quality of Life
METs: Metabolic EquivalenTs
MID: Minimally Important Difference
MRI: Magnetic Resonance Imaging
MVPA: Moderate-to-Vigorous Physical Activity
OR: Odds Ratios
PedsQL: Pediatric Quality of Life
PREVIEW: The Prospective Evaluation of Sport Activity and the Development of Femoroacetabular Impingement in the Adolescent Hip
ROM: Range Of Motion
SD: Standard Deviation
VIBE: Volumetric Interpolated Breath-hold Examination
